# A Psycho-Dynamically Oriented Reflection on Early Sexual Relationships in Pre-Adolescents

**DOI:** 10.3390/children10071270

**Published:** 2023-07-23

**Authors:** Luca Cerniglia, Silvia Cimino

**Affiliations:** 1 Faculty of Psychology, International Telematic University Uninettuno, 00186 Rome, Italy; 2 Department of Dynamic, Clinical and Health Psychology, Sapienza University of Rome, 00185 Rome, Italy; silvia.cimino@uniroma1.it

Pre-adolescence is generally considered a period of change during which sexual energy remains latent before gradually beginning to express itself in adolescence and later in life [1]. However, in recent years, the average age for youths’ first sexual intercourse has dramatically decreased. Globally, over 20% of fourteen year olds have already initiated sexual relationships and engaged in multiple partners within a short time span, and the age of sexual initiation tends to be even lower in most countries [2]. Social media has been suggested to play a role in this trend, as it allows teenagers to access various kinds of information, including implicit sexual content. This exposure can lead to misconceptions and access to pornographic material, encouraging early sexual behavior. Furthermore, social media provides opportunities for unrestricted interactions, which can lead to a cascade of consequences, starting with explicit conversations, video calls, and eventually, in-person sexual encounters [3].

Research has posited that the earlier sexual intercourse begins, the more potential risks it entails, and early sexual initiation serves as a negative indicator of sexual health [4]. Moreover, a premature sexual debut may endanger the emotion regulation processes and the emotional/behavioral functioning youths gain through the interactions with their caregivers during childhood [5,6,7].

While there are widely recognized risks associated with early and promiscuous sexual relationships during early adolescence [8], we believe that it is essential to have a psycho-dynamically oriented discussion in this area to reflect on aspects directly connected with the structuring and functioning of young minds. Although the current scientific literature mostly addresses adolescents’ brain maturation [9], the development of sexuality in young individuals and its impact on their mental functioning is often overlooked and disregarded, being considered an outdated and dusty inheritance. We believe, instead, that the voices of authoritative scholars should still be heard.

For instance, Greenacre [10] emphasized that a child’s encounter with themes related to sexuality, whether in their environment or fantasies, shapes their mind’s development and their ability to relate to others. However, when these experiences are particularly disturbing, especially if they occur too early, very primitive defense mechanisms like denial and repression can be triggered. This can lead to significant alterations in reality testing and perception, resulting in the construction of a “defensive wall”, which the author likened to a dam holding back the excessive stimuli from the environment. This defensive response is similar to what individuals with autism put in place to keep a distance from an environment they perceive as intrusively intense [11].

According to Greenacre, pre-adolescents cannot fully process perceptions and sensations related to adult sexuality and the associated fantasies. Only much later, after successive developmental stages have been reached, can these early experiences be given meaning, lying somewhere between the mental and physical realms. This process fundamentally structures the mind and influences the individual’s relationship with reality. If the path towards mature sexuality is fraught with challenges, including excessive exposure to sexual stimuli, youths may struggle to establish reciprocal and balanced relationships.

Understanding real or fantasized aspects related to sexuality concerning relationships with others and the essential transition towards comprehending three-dimensionality is crucial. This understanding is key for relating to individuals different from oneself. If young people encounter acted-out sexuality prematurely instead of engaging in age-appropriate fantasies and behaviors during the latter stages of adolescence, there is a risk of being unable to invest emotionally in themselves and genuinely connect with others.
